# Adsorption of aqueous Cd(II) and Pb(II) on activated carbon nanopores prepared by chemical activation of doum palm shell

**DOI:** 10.1186/s40064-015-1256-4

**Published:** 2015-08-28

**Authors:** Umar Ibrahim Gaya, Emmanuel Otene, Abdul Halim Abdullah

**Affiliations:** Department of Pure and Industrial Chemistry, Bayero University Kano, Kano, 700241 Kano State Nigeria; Department of Chemistry, Universiti Putra Malaysia, 43400 Serdang, Selangor D.E. Malaysia; Advanced Material and Nanotechnology Laboratory, Institute of Advanced Technology, Universiti Putra Malaysia, 43400 Serdang, Selangor D.E. Malaysia

**Keywords:** Adsorption, Lead, Cadmium, Activated carbon, Doum palm, Mesoporous

## Abstract

Non-uniformly sized activated carbons were derived from doum palm shell, a new precursor, by carbonization in air and activation using KOH, NaOH and ZnCl_2_. The activated carbon fibres were characterised by X-ray diffraction, N_2_ adsorption–desorption, scanning electron microscopy, particle size analysis and evaluated for Cd(II) and Pb(II) removal. The 40–50 nm size, less graphitic, mesoporous NaOH activated carbon yielded high adsorption efficiency, pointing largely to the influence surface area. The performance of the KOH based activated carbon was arguably explained for the first time in terms of crystallinity. The efficiencies of the mesoporous ZnCl_2_-formulated activated carbon diminished due to the presence of larger particles. Batch adsorption of divalent metals revealed dependence on adsorbent dose, agitation time, pH and adsorbate concentrations with high adsorption efficiencies at optimum operating parameters. The equilibrium profiles fitted Langmuir and Freundlich isotherms, and kinetics favoured pseudo-second order model. The study demonstrated the practicability of the removal of alarming levels of cadmium and lead ions from industrial effluents.

## Background

At the global level, heavy metal may enter the environment mainly through uncontrolled anthropogenic fluxes related to mining, refining, plating, ammunition, storage cells, metal smelting and finishing, engine exhausts, industrial emissions and effluents, heavy-metal enrichment of agricultural products, sludge and water reuse. The potential health risk of these metals is mostly associated with exposures to arsenic, cadmium, lead and mercury (Järup 2003). In particular, cadmium and lead ions have long been recognised as hazardous heavy metal pollutants that cannot in anyway be tolerated (Ferm and Carpenter 1967), due to their non-biodegradability, teratogenicity, and latent poisoning (Young 2003; Piskorová et al. 2003).

In today’s society, the quest to remove heavy metals from both industrial and drinking water has heightened proportional interest in the possible water treatment technologies. Of these techniques, adsorption has been considered a good option to at least remove the heavy metals and lessen human’s chances of affliction. Undoubtedly, activated carbon stands in the forefront of the full line of adsorbents (Demirbas 2008). The growing interest in this material is motivated by its favourable surface properties, uniformity of adsorption, and consequently exceptional adsorption effect. In addition, this material is relatively cost effective compared to other inorganic adsorbents such as zeolites.

In the search for efficient, low-cost or ready available activated carbon, several precursors have been investigated. Mobilisation from bagasse pitch, a residual of sugarcane extraction composing largely of cellulose, pentosan, and lignin, realised the removal of Cd^2+^ at record high level efficiency (100 %) as pH exceeds 8.0 (Mohan and Singh 2002). However, the efficiency of activated carbon may be ion dependent. For instance, apricot stone based activated carbon showed high performance in the removal of Cd^2+^ but performed poorly in the removal of Pb^2+^ (Kobya et al. 2005). In our laboratories, we aimed to study the performance of some chemically activated doum palm shell preparations in the adsorptive removal of Cd^2+^ and Pb^2+^ ions from stimulated and industrial effluents. To the best of our knowledge, such form of activated carbon prepared by chemical activation is used for the first time in adsorption study. It is worth mentioning however that Nwosu et al. (2009) have prepared activated carbon from doum palm shells by physical method (oxidation in air, activation in CO_2_ at 840 °C) but these workers limited their study only to the characterisation of surface functionalities (phenolic, lactones), pore size and surface charges of the material. As part of our objectives, we intend to study the effect of operating variables using stimulated Cd^2+^ and Pb^2+^ waters and to treat industrial effluents using the optimised parameters. We also undertake to characterize the surface dependence of the performance of the prepared activated carbons.

## Methods

### Materials

Zinc chloride (97 %) was obtained from BDH, NaOH (97 %) was supplied by Qualikems. During the preparation of KOH (85 %; Reidel-deHaen) compensation for purity was made. Stock solutions of Cd^2+^ and Pb^2+^ were prepared from their nitrates using deionised water. The precursor for activated carbons was doum palm shell. The fruit was collected from the doum palm trees (*Hyphaene thebaica* L. Mart) located in the wild, of Janguza barrack in Gwarzo L.G.A., Kano during dry season (November–December, 2012).

### Experimental details

#### Preparation of activated carbon

Primarily, doum palm shells were scraped and washed to remove the surface husk and dirt, and dried in the oven overnight at 105 °C. The dried doum palm shells were cut, ground and segregated to granular mesh size (1–2 mm). The preparation of activated carbon was largely guided by the method of Tan et al. (2008). Departing from Tan et al. method however, this material was charred at 500 °C for 3 h. The resulting char was impregnated with solid KOH, NaOH and ZnCl_2_ in three separate beakers, at impregnation ratio (*Ir*) of 1:3, defined as follows:

1 $$Ir = \frac{{W_{AA}^{{}} }}{{W_{Char} }}$$

where *W*_*AA*_ is the dry weight (g) of the activating agent, *W*_Char_ the dry weight (g) of char.

Subsequently, deionised water was added to dissolve the activating agent in each mixture, followed by dehydration overnight at 105 °C and further thermal treatment at 500 °C for 3 h. The resulting materials were then cooled and washed with hot deionised water and 0.1 mol/l hydrochloric acid until the pH the filtrate was nearly 7. The activated carbons thus obtained were finally smoothened, stored in well fitted air tight containers and properly labelled KOH-AC, NaOH-AC and ZnCl_2_-AC for experimental investigations. At the same time, the regular activated carbon (regular AC) was prepared by physical procedure, without the addition of the chemical activating agents.

#### Adsorbent characterisation

Surface morphology of the activated carbons (KOH-AC, NaOH-AC, ZnCl_2_-AC and regular AC) was imaged by means of Phenom electron microscope, ProX, MVE 1329F. X-ray diffractogram were recorded on Ital Structure APD2000 X-ray diffractometer. The analysis was performed using Cu Kα radiation source (λ = 1.5406 Å). Adsorption–desorption isotherms were determined using Belsorp Mini II supplied by Bel, Japan. The sorptometer was degassed at 150 °C, except for the regular AC which was degassed at 200 °C. Particle size analysis was performed in triplicates on Nano S Malvern nanosizer and the average particle size was computed. Prior to measurement, the activated carbon samples were dispersed in deionised water by sonication.

#### Moisture content

Ash and moisture content were determined by weight difference (AOAC 1990). For ash content, 5 g of activated carbon was heated in a crucible at temperature of 80 °C for 3 h, cooled in desiccators and then weighed. Sample was heated for the second time at 105 °C for 30 min, cooled in desiccator and weighed again. The procedure was repeated several times at the same temperature for 15 min until a constant weight was obtained. The percentage moisture content of each sample was calculated from using the following equation:

2 $${\text{Moisture content }}(\%) \, = \,\frac{{W_{1} - W_{3} }}{{W_{2} - W_{1} }} \times \, 100$$

The KOH-AC, NaOH-AC, ZnCl_2_-AC and regular AC contain moisture up to 4.4, 5.7, 5.3 and 4.8 % respectively.

In the determination of ash content, activated carbon (1 g) was placed in a crucible of known weight and then heated at of 500 °C for 3 h. The sample was cooled in desiccator and weighed. The ash content of each sample was calculated from the weight of sample before and after heating as follows:

3 $${\text{Ash content }}(\% ) \, = \, \frac{{W_{2} - W_{3} }}{{W_{2} - W_{1} }} \times \, 100$$

where, *W*_1_ is the weight of crucible, *W*_2_ is the initial weight of crucible with sample and *W*_3_ is the final weight of crucible with sample. The ash content of the KOH-AC, NaOH-AC, ZnCl_2_-AC and regular AC was 10.9, 7.6, 8.2 and 11.2 % respectively.

#### Adsorption experiment

The activated carbon powders (KOH-AC, NaOH-AC, ZnCl_2_-AC and regular AC) were used to adsorb Pb^2+^ and Cd^2+^ from stimulated waste water. Batch experiments were basically performed in thermostated shakers at 30 °C to investigate the effect of pH, adsorbent dosage, contact time and initial concentration of Cd^2+^ or Pb^2+^ ions on adsorption over doum palm shell adsorbents. After the flasks were agitated, the content of each flask was removed, filtered and the filtrate analysed by atomic absorption spectrometer (AAS). The metal concentration (mg/g) retained on the adsorbent phase (*q*_*e*_) and the removal efficiency (%) of the adsorbent preparations were calculated using the following equations respectively:

4 $$q_{e} = \frac{{C_{o} - C_{e} }}{m} \, \times \, V$$

5 $${\text{Removal efficiency }}(\%) = \frac{{C_{o} - C_{e} }}{{C_{o} }} \, \times { 100}$$

where *C*_*o*_ and *C*_*e*_ are the concentrations (mg/l) of Cd^2+^ and Pb^2+^ before and after adsorption respectively, *V* (ml) is the volume of the Cd^2+^ or Pb^2+^ and *m* (g) is the mass of the adsorbent.

##### Effect of operating variables

The effect of operating variables on the adsorption of Cd^2+^ or Pb^2+^ was studied using the synthetic heavy metal solutions and in each case the optimum condition is surmised. The influence of agitation time on the adsorption of Cd^2+^ or Pb^2+^ (50 ml of 50 mg/l) was studied in a batch system containing 2.5 g activated carbon (KOH-AC, NaOH-AC, ZnCl_2_-AC or regular AC). The flasks were corked and agitated at 350 rpm within different contact times (0, 20, 40, 60, 80 and 100 min) and aliquots were taken, filtered and analysed using AAS.

In order to determine the effect of adsorbent concentration, stimulated solution of Cd^2+^ or Pb^2+^ (50 ml) was added into the batch reactor containing 0.5–2.5 g of KOH-AC, NaOH-AC, ZnCl_2_-AC or regular AC, and agitated at 350 rpm between 40 and 80 min depending on the type on the equilibrium agitation time adsorbent. The contents in the flasks were filtered and each filtrate was analysed using AAS. On the other hand, the influence of adsorbate concentration (Cd^2+^ or Pb^2+^ mg/l) was studied between concentrations of 40–80 mg/l. Fifty milliliter of heavy metal solution was agitated until equilibrium was attained. After the adsorption, the residual suspension was filtered to remove the doum palm stone and absorbance of filtrate was measured using atomic absorption spectrophotometer. The optimum conditions from the above experiments were maintained, but the pH of each five set of solutions were adjusted between 5 and 7 (through intervals of 0.5) using 0.1 mol/l HCl and 0.1 mol/l NaOH. The content of each flask was filtered, and the filtrate was analysed using AAS.

##### Equilibrium and kinetic adsorption parameters

The isotherms often subscribed to, in order to model the equilibrium of heavy metal adsorption include the Langmuir, Freundlich and Temkin. In this study equilibrium profiles, or adsorption isotherms were fitted into the linear form of Freundlich, Langmuir and Temkin isotherms:

6 $$\log q_{e} = \log K_{F} + \frac{1}{n}\log C_{e}$$

7 $$\frac{1}{{q_{e} }} = \frac{1}{{q_{m} }} + \frac{1}{{C_{e} K_{L} q_{m} }}$$

8 $$q_{e} = B\log A + B\log C_{e}$$

where *K*_*F*_ is the Freundlich adsorption constant, *K*_*L*_ is the Langmuir adsorption constant, *q*_*m*_ is the maximum concentration of heavy metals adsorbed and A and B are constants.

Kinetic study was performed using 50 ml adsorbate solution, in the concentration range of 40–80 mg/l and in the presence of 2.5 g of activated carbon. Samples were taken at some intervals of contact time (0, 20, 40, 60, 80 and 100 min), filtered and the filtrate was analysed using AAS.

#### Effluents sampling and analysis

Ten representative effluent samples, were collected mainly from tanning and textile industries of the Sharada industrial estate of Kano (lat. 11°30^″^N, long. 8°38^″^E, alt. ~485 m). The identity of these manufacturing industries is herein replaced by letters A–J.

Effluents were digested by the APHA (1985) method as follows. Concentrated HNO_3_ (5 ml) was added to 100 ml of effluent in a beaker which was covered with watch glass and concentrated to 20 ml in fume hood. Equal volume of the HNO_3_ (5 ml) was added to the mixture and heated gently to easily reflux, until a light, clear solution was obtained. The beaker and the watch glass were washed with distilled water and transferred into 100 ml volumetric flask and diluted to the mark with distilled water. The cadmium and lead concentration of the effluent samples and the standard aqueous solutions was determined using 210VGP Buck Scientific, atomic absorption spectrophotometer (AAS). The analysis was performed in triplicates and the levels of cadmium and lead before adsorption experiments were recorded (Table 1). These levels of these heavy metals were then treated using the optimum doses of activated carbon.

**Table 1:** Levels of ${\text{Cd}}_{{({\text{aq}})}}^{ 2+ }$ and ${\text{Pb}}_{{({\text{aq}})}}^{ 2+ }$ in the industrial effluents before adsorptive treatment

Sample^a^	Cd^2+^ (mg/l)^b^	Pb^2+^ (mg/l)^b^
A (textile industry)	0.66 ± 0.01	0.13 ± 0.01
B (tanning industry)	0.69 ± 0.01	0.39 ± 0.03
C (tanning industry)	0.82 ± 0.02	0.52 ± 0.02
D (packaging materials industry)	0.98 ± 0.01	0.92 ± 0.01
E (insecticide industry)	1.02 ± 0.03	1.18 ± 0.01
F (textile industry)	1.08 ± 0.01	1.32 ± 0.02
G (tanning industry)	1.26 ± 0.02	2.09 ± 0.01
H (textile)	1.30 ± 0.04	2.87 ± 0.02
I (tanning industry)	1.42 ± 0.02	3.26 ± 0.03
J (tannery)	2.06 ± 0.05	4.57 ± 0.03

^a^The names of these manufacturing companies are reserved by the authors

^b^Mean + standard deviation

## Results and discussion

### Effect of contact time

**Fig. 1:**
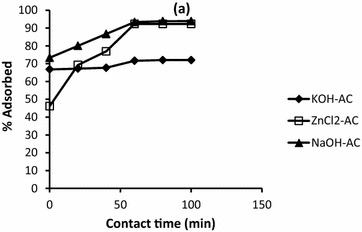
Effect of contact time on the adsorption of Pb^2+^ by 50 mg/l ZnCl_2_, KOH and NaOH activated, and regular activated doum palm shell

Basically, the efficiency of adsorption strongly depends upon the time of adsorption. So the effect of contact time in the adsorption of cadmium and lead was studied. In the case of lead, the results of the study are depicted by Fig. 1. From the figure, the equilibrium adsorption was attained after about 60 min for all the activated carbon powders with adsorption capacities of 71.62, 93.34 and 92.31 % for KOH-AC, NaOH-AC and ZnCl_2_-AC, respectively.

A general observation was that before equilibrium, there is rise in the removal efficiency of both Cd^2+^ and Pb^2+^ with increasing contact time which is universally true for good adsorbents (Sekar et al. 2004; Wang et al. 2010; Liao et al. 2011). In a similar manner to that of lead, the equilibrium for cadmium adsorption was attained within short time, typically 60 min.

### Effect of initial concentration

**Fig. 2:**
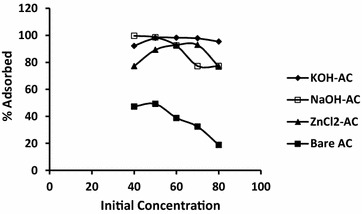
Influence of Cd^2+^ concentration on the adsorption capacity of the activated carbon adsorbents

The effect of initial heavy metal concentration was studied within the range of 40–80 mg/l. The results of the study are shown in Fig. 2. From the figure, the removal efficiency of Pb^2+^ by KOH-AC and ZnCl_2_-AC increases with increasing presence of the adsorbate due to the availability of adsorption sites on the activated carbons. However, as the lead concentration becomes exceedingly high, in most cases beyond the optimum concentration (50 mg/l), the removal efficiency declines. This concentration was selected as optimum and used in the study to investigate the effect of contact time, adsorbent dosage and pH. It may be noted that the efficiency of adsorption was generally high (>70 %) with all of the chemically activated carbons.

It is generally accepted that the mechanism for metal removal is related to the surface properties of activated carbons. Specifically speaking, regular doum palm activated carbon, prepared by CO_2_ activation, showed the possibility of exhibiting varied pore sizes, large quantity of oxygen containing functionalities (phenolic-OH/lactonic=O up to 8.294 mmol H^+^ equiv./g C) and total negative surface charge (up to 5.636 mmol H^+^ equiv./g C) (Nwosu et al. 2009) which synergistically can provide active sites for adsorption of heavy metal salts. Improvement in the adsorption efficiency with the activated carbons (NaOH-AC, KOH-AC, ZnCl_2_-AC) as shown (Fig. 2) may therefore be linked to the improved surface properties resulting from chemical activation.

### Effect of adsorbent dosage

**Fig. 3:**
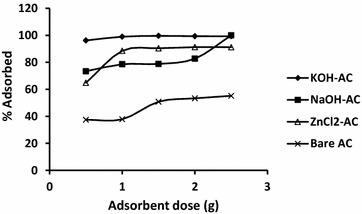
Effect of adsorbent dosage on the adsorption of Cd^2+^ by ZnCl_2_, KOH and NaOH AC, and the regular activated doum palm powder

The effect of adsorbent dosage was investigated using 50 mg/l initial adsorbate concentration with adsorbent concentration within 0.5 g/50 ml to 2.5 g/50 ml. The results of the investigations of cadmium removal are displayed in Fig. 3. At the initial stage generally, there is gradual increase in removal efficiency of Cd^2+^ with corresponding increase in adsorbate concentration which may be attributed to the increasing presence of adsorption sites. However, as the binding sites of the activated carbons get saturated the curves level off due to the independence of the adsorption on concentration of the adsorbate. It is evidently clear from the figure that with KOH-AC remarkable adsorption was obtained within the range of adsorbent dosage used in the study. Accordingly, the optimum doses for effective cadmium removal over ZnCl_2_-AC, KOH-AC, NaOH-AC and regular AC were 1.5, 1.0, 2.5, and 1.5 g (with adsorption capacities 99.82, 99.52, 100 and 66.84 %), respectively.

**Fig. 4:**
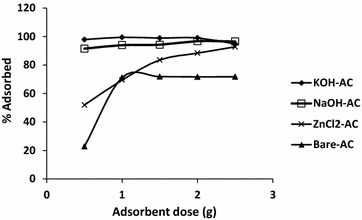
Effect of adsorbent dosage on the adsorption of Pb^2+^ by ZnCl_2_, KOH and NaOH activated carbons and bare activated carbons

On the other hand, Fig. 4 shows the effect of adsorbent amounts on the adsorption of Pb^2+^. The trends in the outstanding performance of KOH-AC and NaOH-AC did not vary significantly from the case of Cd^2+^, in the fact that the adsorption capacity below the optimum adsorbent dosage is reached. The performance of the activated carbons ZnCl_2_-AC, KOH-AC, NaOH-AC and regular AC, at the optimum adsorbent doses (1.5, 1.0, 1.0, and 1.0 g) were 99.92, 99.60, 99.96 and 82.52 %) respectively. On the whole, it can be generalised that the quantity of adsorbent was found to influence the adsorption efficiency negatively except at the optimum doses. In addition, because the regular AC yielded the lowest adsorption capacity for Cd^2+^ and Pb^2+^ (55.22 and 71.88 %, respectively) the remarkable applicability of NaOH and KOH activation for carbonaceous materials is reassured.

### Influence of surface properties of the adsorbent

**Fig. 5:**
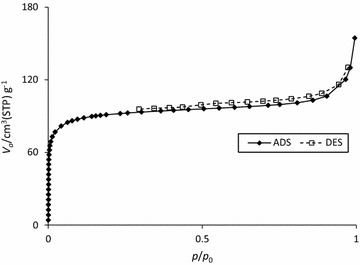
Adsorption–desorption isotherm of NaOH-AC at 77 K

In order to find the factor responsible for high efficiency of adsorption with KOH-AC and NaOH-AC, adsorption–desorption experiments were conducted. A marked difference in porosity of the activated carbons was readily noticed. The N_2_ adsorption–desorption isotherm of NaOH-AC (Fig. 5) was of type IV, which is characteristic of mesoporous material based on the Bauneuer–Deming–Deming–Teller (BDDT) interpretation. The characteristic hysteresis loop of this type of porous material can be easily seen from the figure.

**Fig. 6:**
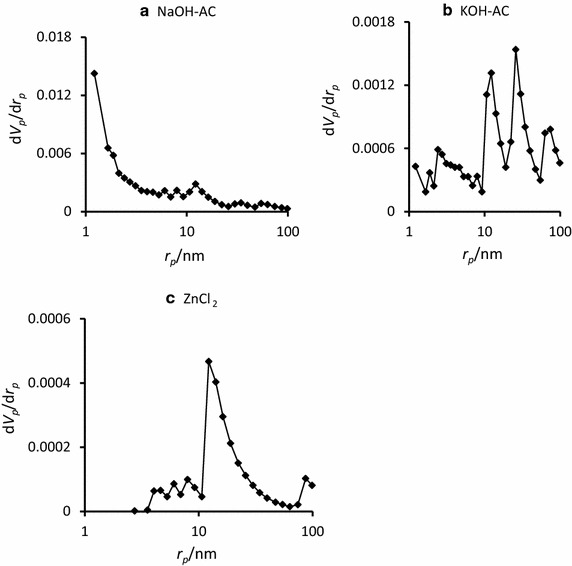
The BJH pore size distribution of NaOH-AC (**a**), KOH-AC (**b**) and the regular AC (**c**)

Obviously, the high adsorption capacity of the NaOH-AC (S_BET_ = 226.02 m^2^/g) compared to the other modified activated carbon adsorbents (S_BET__(KOH-AC)_ = 5.41 m^2^/g and ${\text{S}}_{{{\text{BET}}({\text{ZnCl}}_{2} - {\text{AC}})}} = 0. 8 4 {\text{m}}^{ 2} /{\text{g}}$ ) is surface area-driven. To corroborate this view the pore size distribution was assessed by the Barrett–Joyner–Halenda (BJH) method (Fig. 6). The results were positive as NaOH-AC showed the highest porosity (average pore volume V_p_ = 0.096 cm^3^/g; estimated at p/p_o_ = 0.990) compared to the KOH-AC and ZnCl_2_-AC (V_p_ = 0.068 and 0.008 cm^3^/g). Generally, the activated carbons show varied pore size distributions depending upon the activating agent. Basically, the International Union of Pure and Applied Chemistry (IUPAC) has described micropores, mesopores and macropores with distinctive pore diameters of ≤2, 2–50 and ≥50 nm, respectively. From Fig. 6a it can be concluded that the NaOH-AC consists predominantly of mesopores. The average mesopore diameter of this activated carbon was determined to be 3.99 nm. Interestingly, the ZnCl_2_-AC is also mesoporous (average pore diameter = 40.2 nm).

**Fig. 7:**
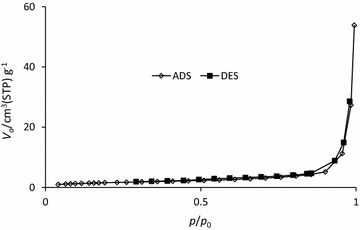
Nitrogen adsorption–desorption isotherm for KOH-AC, at 77 K

The KOH-AC isotherm displays a type III macropore (Fig. 7) and this can be confirmed by the average pore diameter (50.12 nm). However, the remarkable performance of this activated carbon could not be attributed to surface area because the S_BET_ is low.

**Fig. 8:**
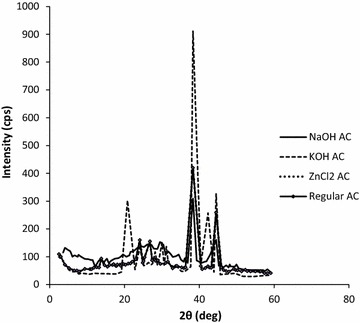
X-ray diffraction patterns of NaOH-AC, KOH-AC, ZnCl_2_-AC and regular AC

In order to provide plausible explanation for the efficiency of KOH-AC, X-ray diffraction (XRD) analysis was conducted. An overlay of the diffractograms for the activated carbons is shown in Fig. 8. The prominent diffraction peaks, common to all the activated carbons were observed at 2θ = 24.1°, 38.3°, and 44.5°. To aid the foregoing discussion, a summary of the scattering angles for each activated carbon in a tabular form is presented (Table 2). Obviously, heat treatment has graphitized the activated carbons as indicated by the characteristic faces of the graphite lattice. All the activated carbons show the characteristic diffraction peaks at 2θ = 24.1° for the structural 002 reflection of the carbon layer, as commonly interpreted for activated carbons (Iijima 1991; Acharya et al. 2009). The peak at 2θ = 42.3° is for the 10 reflection, an orientation resulting from a merger of 101 and 100 (Dandekar et al. 1998). The peak at 44.5°, which is common to all of the activated carbon powders, corresponds to the sp^3^ lattice reflections.

**Table 2:** List of scattering angles of the as prepared activated carbons

Activated carbon	Observed scattering angle, 2θ°
NaOH-AC	26.1°, 38.3°, 44.5°
KOH-AC	20.8°, 24.1°, 28.2°, 30°, 38.3°, 42.3°, 44.5°
ZnCl_2_-AC	13.8°, 24.1°, 26.8°, 38.3°, 44.5°
Regular AC	13.8°, 24.1°, 26.8°, 38.3°, 44.5°

Generally, the higher the intensity of XRD peaks of a material the higher the crystallite sizes. The diffraction peaks of the KOH-AC are the sharpest and most intense due to the increased crystallinity, particle size and purity. The crystallinity is in the order KOH-AC > ZnCl_2_-AC = regular AC > NaOH-AC. The peak pattern of the ZnCl_2_-AC and the regular AC is exactly the same. From the broadening of the diffraction peaks with respect to NaOH-AC, a relatively high amorphous content along with high porosity could easily be confirmed. The shift in the diffraction peaks is related to the change in crystallinity which broadens or sharpens the peaks respectively.

**Fig. 9:**
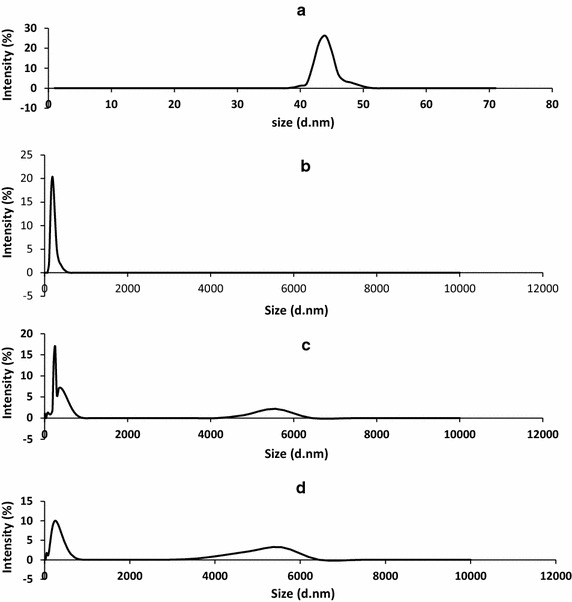
Particle size distribution of NaOH-AC (**a**), KOH-AC (**b**), ZnCl_2_-AC (**c**) and regular AC (**d**)

The size distribution of the particles was analysed using particle sizer. The results are depicted by Fig. 9. From the insets (a) and (b) both KOH-AC and NaOH-AC showed unimodal particle size distribution with average particle size (nm) peaking at 190.10 ± 0.00 and 43.82 ± 0.00, respectively. The pore size range of the KOH-AC was 0.4–458.7 nm while the NaOH-AC was in the rage of 31–51 nm. Differently, bimodal pore size distribution was obtained for the ZnCl_2_-AC and regular AC, falling amazingly within the same ranges (0.4–825 and 4145–6439 nm). These activated carbons differ only in the peak symmetry as the first peak of the ZnCl_2_-AC doubled at 342 nm (Fig. 9c) as a result of density of particles with those sizes whereas such peak doubling was not observed with the regular AC (Fig. 9d). From the figures, the lower performance of the ZnCl_2_-AC and regular AC as compared to the other adsorbents can be traced to the presence of larger particle sizes in these activated carbons (in the order of microns), which provide lower surface area as compared to KOH-AC and NaOH-AC.

**Fig. 10:**
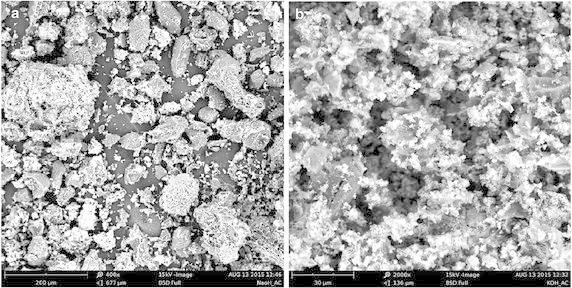
Scanning electron micrographs of the surface of **a** NaOH-AC, **b** KOH-AC

The high crystallinity of the KOH-AC particles prompted us to image surface morphologies of the activated carbons using scanning electron microscopy (SEM) and visualise possible variations. Figure 10 displays the scanning electron micrographs of NaOH-AC and KOH-AC. Both SEM images indicate rudimentary surfaces similar to those of activated carbon powder from cashew nut shell (Jung et al. 2014), which may be attributed to incomplete release of volatiles. In particular, the NaOH-AC shows the presence of tiny pores, which appear to be in the course of development. These pores are not visible in the KOH-AC which perhaps account for its relatively lower surface area.

Lastly, the ash content of adsorbent can provide considerable information on adsorption capacity. The lower the ash content of activated carbon the better its adsorption capacity. Whereas NaOH-AC had the lowest ash content (8.2 %), the regular AC has the highest amount of this property (11.2 %) which further speaks for its lower performance compared to the latter.

### Effect of pH

**Fig. 11:**
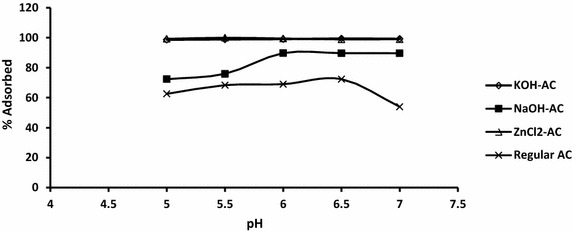
Effect of pH on the adsorption of Cd^2+^ by ZnCl_2_, KOH and NaOH modified, and unmodified doum palm stone

The pH of the aqueous suspension of adsorbent is an important parameter that may control the adsorption of metals (Chen et al. 2010). It can be seen from Fig. 11 that for the regular AC and the NaOH-AC, low removal capacity of cadmium was observed at pH 5 which improved fairly as acidity is increased to pH 6. With the regular AC decrease in the adsorption efficiency for cadmium ions is observed at pH > 6.5. In general, the drastic reduction in the adsorption of cadmium(II) ions and lead(II) ions on in the suspensions of ZnCl_2_, KOH, NaOH activated doum palm stone and precursor respectively, within the pH range of this study (pH 5–pH 7) may be due to the precipitation of hydroxides as predicted by the Pourbaix diagram. In fact Pb^2+^ is known to precipitate as Pb(OH)_2_ above pH 6.7 (Momčilović et al. 2011). In the case of cadmium ion, the increase in removal can be attributed to the low concentration of positive surface charges such as H^+^ which would lessen the repulsion of the positive metal ion and enhance adsorptivity (Kadirvelu and Namasivayam 2003). Based on Fig. 11, the optimum pH for the removal of Cd^2+^ over NaOH-AC can be adjudged to be 5.5.

In “Effluents sampling and analysis”, the levels of divalent Cd and Pb in the effluents from several industries have been tabulated. The values of Cd^2+^ in the industrial effluents were all objectionable, being well above the recent WHO guidelines (0.003 mg/l) (WHO 2006), so they can be used to assess the suitability of the activated carbon adsorbents for practical use. The removal of this metal from the industrial effluents was demonstrated using NaOH-AC at the optimum conditions of pH 5.5, adsorbent dose (1.5 g/50 ml) and initial cadmium concentration (40 mg/l). The results of the adsorption study are shown in Table 3.

**Table 3:** Values of adsorption parameters for cadmium

Samples	Cd^2+^ (mg/l)	C_e_ (mg/l)	q_e_ (mg/l)	q_t_ (mg/g)	% Adsorbed
A	0.66	0.04	0.62	0.0083	93.94
B	0.69	0.02	0.67	0.0089	97.10
C	0.82	0.07	0.75	0.0100	91.46
D	0.98	0.05	0.93	0.0124	94.89
E	1.02	0.10	0.92	0.0123	90.10
F	1.08	0.08	1.00	0.0133	92.59
G	1.26	0.22	1.04	0.0139	82.54
H	1.30	0.01	1.29	0.0172	99.23
I	1.42	0.09	1.33	0.0177	93.66
J	2.06	0.44	1.62	0.0216	78.64

Optimum operating conditions; adsorbate concentration = 40 mg/l, pH = 5.5, temperature = 30 °C and adsorbent dosage = 1.5 g/50 ml

Similarly, the Pb^2+^ levels in the industrial effluents cannot be tolerated as they exceed the limits of WHO (2006) (0.01 mg/l). So, the performance of NaOH-AC in the treatment of these practical samples was evaluated using the optimum conditions achieved with the stimulated samples (Table 4). The results were remarkable as the least removal efficiency was 73.48 %. Generally, there is no correlation between the adsorption capacity of the NaOH-AC of lead and cadmium (correlation coefficient R^2^ = −0.24714), which shows the variation in the affinity of the adsorbents for the target metals.

**Table 4:** Values of adsorption parameters for lead

Sample	Pb^2+^ (mg/l)	C_e_ (mg/l)	q_e_ (mg/l)	q_t_ (mg/g)	% Adsorbed
A	0.13	0.02	0.11	0.0022	84.62
B	0.39	0.08	0.31	0.0062	79.49
C	0.52	0.01	0.51	0.0102	98.08
D	0.92	0.19	0.73	0.0146	79.35
E	1.18	0.30	0.88	0.0176	74.58
F	1.32	0.35	0.97	0.0194	73.48
G	2.09	0.22	1.87	0.0374	89.47
H	2.87	0.04	2.83	0.0566	98.61
I	3.26	0.27	2.99	0.0598	91.72
J	4.57	0.08	4.49	0.0898	98.25

Optimum operating conditions: [Pb^2+^] = 50 mg/l, pH = 6.0, temp = 30 °C and adsorbent dosage = 1.0 g/50 ml

### Adsorption isotherms, kinetics and mechanism

Among the many adsorption isotherms used to model the amount of solute adsorbed per unit of adsorbent as a function of equilibrium concentration in the bulk solution Freundlich, Langmuir and Temkin were evaluated at constant temperature. In this study, consistence was only obtained with both Freundlich and Langmuir isotherms and their parameters for cadmium and lead adsorption are presented in Table 5.

**Table 5:** Langmuir and Freundlich equilibrium parameters for the adsorption of lead and cadmium on activated carbons

Adsorbent	Metal	Langmuir parameters				Freundlich parameters		
		q_max_ (mg/g)	K_L_ (l/mg)	R_L_	R^2^	N	K_F_ (l/mg)	R^2^
NaOH-AC	Cd(II)	100	2.01 × 10^−4^	0.999	0.996	1.00	0.02	1.00
	Pb(II)	500	4.01 × 10^−5^	0.998	1.000	1.00	0.02	1.00
KOH-AC	Cd(II)	1000	2.00 × 10^−4^	0.999	1.000	1.00	0.02	1.00
		333	6.03 × 10^−5^	0.997	1.000	1.00	0.02	1.00
ZnCl_2_-AC	Cd(II)	125	1.59 × 10^−4^	0.992	1.000	0.99	0.02	0.99
	Pb(II)	250	8.04 × 10^−5^	0.996	1.000	1.00	0.02	1.00
Regular AC	Cd(II)	−125	1.60 × 10^−4^	0.992	1.000	1.00	0.02	1.00
	Pb(II)	167	1.20 × 10^−3^	0.943	1.000	1.00	0.02	1.00

It would be seen from the table that the Freundlich and Langmuir isotherms are linear with correlation coefficient R^2^ in the range of 0.943–1.000. Agreement with both of these isotherms was previously reported for lead adsorption on carbon nanotube (Li et al. 2003) and potassium hydrogen phthalate adsorption on TiO_2_ (Valente et al. 2006). The separation factor *R*_*L*_ is usually employed to describe the favourability of the adsorption. It is based on the Langmuir adsorption constant:

9 $$R_{L} = \frac{1}{{1 + K_{L} C_{o} }}$$

where *K*_*L*_ (l/mg) is the Langmuir isotherm constant (obtainable from Eq. (7)), and *C*_*o*_ (mg/l) is the initial metal (II) concentration. If *R*_*L*_ > 0 but <1 the Langmuir isotherm is favorable as it is the case in our study. However, if *R*_*L*_ = 1 or 0, this type of isotherm is unfavourable or linear, respectively. Both the Freundlich isotherm and the Langmuir isotherms are favoured as indicated by the near-unity correlation coefficients.

By far, several kinetic models have been successfully applied to various adsorption processes over biosorbents, which include the Dubinin-Radushkevich, Elovich, zero order, first order, second order, third order equations (Ho et al. 2000). The linear pseudo-second order equation takes the form:

10 $$\frac{t}{{q_{t} }} = \frac{1}{{k_{2} q_{e}^{2} }} + \left( {\frac{1}{{q_{e} }}} \right)t$$

So, the plot of t/q_t_ against t will give a straight line having a slope 1/q_e_ and intercept 1/k_2_q_e_^2^. In the present study, the kinetics of Cd^2+^ and Pb^2+^ adsorption were largely consistent with the pseudo-second order rate equation. The adsorption parameters derived from the pseudo-second order kinetic model are displayed in Table 6.

**Table 6:** Kinetic parameters for Cd^2+^ and Pb^2+^ removal using the home-prepared porous carbons

Adsorbent	Metal	*q*_*e*_ (mg/g)	Pseudo-second order	
			*k*_2_ (g/mg min)	R^2^
KOH-AC	Cd^2+^	0.992	3.83	0.999
KOH-AC	Pb^2+^	0.985	12.61	1.000
NaOH-AC	Pb^2+^	0.993	1.82	0.999

Primarily, the pseudo-second order model is based on the assumption that the rate-determining step may be chemisorptions, capitalizing on the exchange of electrons between adsorbent and adsorbate (Ho et al. 2000; Kula et al. 2008). Our results have favoured this kinetic model for NaOH-AC and KOH-AC, while the rest of the activated carbons did not agree with any monolinear model. This is notwithstanding the fact that the adsorption of Cd^2+^ on activated carbons prepared using ZnCl_2_ activation were earlier found to comply with the pseudo-second kinetic scheme.

Basically, adsorption mechanism has been explained in terms of cation exchange, complexation and diffusion. In porous adsorbents, the rate limiting step may be diffusion. Because of the porosity of the activated carbons used in this study, the effect of interparticle diffusion was investigated utilising the Weber–Morris intraparticle diffusion model:

11 $$q_{t} = k_{{\rm int}} {\text{t}}^{1/2} + {\text{C}}$$

where *k*_int_ is the intraparticle diffusion rate constant. A plot of *q*_*t*_ versus t^½^ (min^½^) should give a straight line with an intercept C and slope *k*_int_ (g/mg min^½^). Our results of the application of this model rule out the influence of the diffusion kinetics, and by implication confirm the predominance of surface chemisorption.

### Thermodynamic studies

Thermodynamic parameters were determined following experiments in temperature range of 303–323 K. Generally, the heterogeneous equilibrium constant *K*_*c*_ for the adsorption equilibria is given by the equation:

12 $$K_{c} = \frac{{C_{s} }}{{C_{e} }}$$

where *C*_*s*_ (mg/l) is the amount of adsorbate in the adsorbed phase and *C*_*e*_ (mg/l) is the equilibrium concentration of solution. The values of *K*_*c*_ for adsorption of Cd(II) and Pb(II) for the various activated carbons used in the study at various temperatures were calculated. The corresponding values of the Gibbs free energy change (∆*G*^*o*^) were calculated using the relation:

$$\Delta G^{0} = \, - RT{ \ln }K_{c}$$

Similarly, those of other thermodynamic functions such as adsorption enthalpy (∆*H*^*o*^) and entropy change (∆*S*^*o*^) by the relation:

13 $$\ln K_{c} = - \frac{{\Delta H^{0} }}{RT} + \frac{{\Delta S^{0} }}{R} .$$

Table 7 shows the values of ∆*G*^*o*^, ∆*H*^*o*^ and ∆*S*^*o*^ determined in this study for the adsorption of Pb^2+^ over the chemically activated carbon fibres. The negative values of the Gibbs free energy showed the thermodynamic favourability of the adsorption processes. Generally speaking, increase in adsorption rate with temperature was observed within the temperature range of the study, which may be attributed to the increase in the mobility and diffusion of ionic species into the pore sites of the activated carbons. These temperatures are not sufficient enough to desorb the adsorbates in the adsorbed phase due to the high adsorption enthalpies predicted (57–100 J/mol) relative to the entropies. This opinion is supported by the applicable pseudo second order kinetic scheme, which predicts chemisorptions.

**Table 7:** Thermodynamic functions of the removal of Pb^2+^ over the chemically activated carbons

Temperature	Functions	KOH-AC	NaOH-AC	ZnCl_2_-AC
303	∆*G* ^*o*^	−3.885	−0.7878	−6.89
308		−4.151	−0.9116	−7.74
313		−4.663	−1.332	−8.17
318		-6.274	−4.0873	−8.58
323		−7.737	−4.7771	−9
	∆*S* ^*o*^	199.5	230.1	100.8
	∆*H* ^*o*^	57.0	69.6	7.0

## Conclusion

In sum, NaOH and KOH activated carbons from doum palm shell are new, efficient adsorbents for the remarkable removal of deleterious levels of cadmium or lead from aqueous environment. The adsorption is limited by pH. In addition, surface properties such as crystallinity, particle size and surface area, were found to show significant influence on the efficiency of the adsorbents. These properties can easily be tuned by the variant of chemical activating agent. The equilibrium adsorption isotherms consistent with the adsorption of the prepared activated carbons were Freundlich and Langmuir. The adsorption is chemisorptions controlled and thermodynamically favourable even at warmer temperatures.
